# Comprehensive Gene Expression Analysis Using Human Induced Pluripotent Stem Cells Derived from Patients with Sleep Bruxism: A Preliminary In Vitro Study

**DOI:** 10.3390/ijms252313141

**Published:** 2024-12-06

**Authors:** Taro Sato, Akihiro Yamaguchi, Mayu Onishi, Yuka Abe, Takahiro Shiga, Kei-ichi Ishikawa, Kazuyoshi Baba, Wado Akamatsu

**Affiliations:** 1 Department of Prosthodontics, Graduate School of Dentistry, Showa University, Ota-ku, Tokyo 145-8515, Japan; 2 Center for Genomic and Regenerative Medicine, Juntendo University School of Medicine, Bunkyo-ku, Tokyo 113-8421, Japan

**Keywords:** sleep bruxism, human induced pluripotent stem cells, transcriptome, calcium signaling

## Abstract

Sleep bruxism (SB) involves involuntary jaw movements during sleep and is potentially caused by motor neuronal hyperexcitability and GABAergic system dysfunction. However, the molecular basis remains unclear. In this study, we aimed to investigate changes in the expression of several genes associated with the pathophysiology of SB. Bulk RNA sequencing (bulk RNA-seq) and single-nucleus RNA sequencing (snRNA-seq) of neurons derived from patient and control human induced pluripotent stem cells (hiPSCs) were performed to comprehensively assess gene expression and cell type-specific alterations, respectively. Bulk RNA-seq revealed significant upregulation of calcium signaling-related genes in SB neurons, including those encoding G protein-coupled receptors and receptor-operated calcium channels. snRNA-seq confirmed the increased expression of *GRIN2B* (an N-methyl-D-aspartate receptor subunit) and *CHRM3* (an M3 muscarinic acetylcholine receptor), particularly in glutamatergic and GABAergic neurons. These alterations were linked to hyperexcitability, with *GRIN2B* contributing to glutamatergic signaling and *CHRM3* contributing to cholinergic signaling. These findings suggest that disrupted calcium signaling and overexpression of *GRIN2B* and *CHRM3* drive neuronal hyperexcitability, providing insight into the pathophysiology of SB. Targeting these pathways may inform therapeutic strategies for SB treatment.

## 1. Introduction

Sleep bruxism (SB) is a sleep-related movement disorder defined as the repetitive activation of the masticatory muscles during sleep, characterized by tooth grinding or clenching according to the International Classification of Sleep Disorders, Third Edition (ICSD-3) [1]. Excessive forces on the masticatory muscles due to SB can lead to various harmful symptoms, including temporomandibular joint disorder, abnormal tooth wear, tooth fractures, periodontal disease, masticatory dysfunction, and facial pain [2]. Numerous factors associated with SB include alcohol consumption, caffeine intake, stress, and central nervous system (CNS) stimulants, such as calcium channel blockers and selective serotonin reuptake inhibitors. Genetic predispositions have been suggested based on twin and family aggregation studies [3,4,5]. Additionally, various sleep disorders, such as insomnia, restless legs syndrome, periodic limb movement disorder, rapid eye movement (REM) sleep behavior disorder, and sleep-related epilepsy [6,7], have been linked to SB [3,4,5].

Dysregulation of rhythmic masticatory muscle activity (RMMA) is characterized by repetitive jaw muscle contractions associated with the sleep–wake state [8,9]. During REM sleep, the activity of the trigeminal motor neuron, which regulates masticatory muscle activity, is typically suppressed by inhibitory neurons [10], such as GABAergic and glycinergic neurons, resulting in minimal muscle activity during sleep [11]. However, during shallow non-REM sleep, RMMA is often associated with micro-arousal (MA), characterized as an arousal event lasting for 3–15 s, accompanied by transient increases in the activities of the central and sympathetic nervous systems during sleep [12]. While healthy individuals do not experience SB even when MA occurs, patients with SB typically exhibit RMMA accompanied by MA in most cases [13,14]. Furthermore, patients with SB may show abnormalities in the GABAergic system of the brainstem network [15,16]. These previous findings suggest that neurotransmitters within the CNS play a role in generating SB and that variations in the sensitivity of CNS neurons to sympathetic activity changes during MA lead to motor neuronal hyperexcitability, potentially contributing to RMMA [10].

We previously generated human induced pluripotent stem cells (hiPSCs) from three patients with SB who met clinical criteria [17] and had the C/C genotype of the rs6313 allele, a single-nucleotide polymorphism (SNP) in the serotonin 2A (5-HT2A) receptor gene (*HTR2A*), which is a risk factor for SB [18]. Because *HTR2A*-positive neurons are expressed in the ventral hindbrain, we developed a protocol to efficiently differentiate hiPSCs into brainstem neurons, which regulate muscle activity during the sleep–wake cycle [19,20]. Furthermore, we revealed that brainstem neurons differentiated from patients with SB exhibited significantly higher excitability than those from healthy controls with the T/T genotype, characterized by increased action potential firing frequency, enhanced gain, and decreased action potential half-duration [20]. However, the factors driving this SB-specific phenotype, the responsible cell populations, and the electrophysiological properties of cells that form neural networks remain unclear. Based on these findings, we hypothesized that the hyperexcitability of brainstem neurons in patients with SB results from dysregulated gene expression influenced by genetic factors, such as SNPs, and alterations in neuronal signaling pathways. These changes are proposed to contribute to RMMA during sleep by affecting the excitability of specific neuronal subtypes, including glutamatergic, GABAergic, and motor neurons.

Although previous studies have highlighted the potential role of genetic factors, neurotransmitters, and neuronal excitability in SB, critical gaps remain in understanding the precise molecular and cellular mechanisms underlying this condition. To date, no study has conducted gene expression analyses using induced pluripotent stem cells (iPSCs) derived from patients with SB, making this a novel approach to investigating the molecular basis of SB. Moreover, the specific neuronal subpopulations contributing to RMMA during sleep and the mechanisms by which dysregulation of gene expression drives hyperexcitability are yet to be elucidated. Addressing these questions is essential for bridging the gap between clinical observations and the underlying neurophysiological processes and identifying potential therapeutic targets.

In this study, we aimed to elucidate the mechanism underlying the dysregulation of hyperactivity in masticatory muscles. We performed bulk RNA sequencing (bulk RNA-seq) and single-nucleus RNA sequencing (snRNA-seq) of neuronal populations differentiated from hiPSCs derived from patients with SB to assess comprehensive gene expression profiles. Additionally, we investigated neural populations and identified genes with variable expression across distinct neuronal clusters.

## 2. Results

### 2.1. Polarization of Gene Expression Patterns in Control and SB Samples

**Figure 1:**
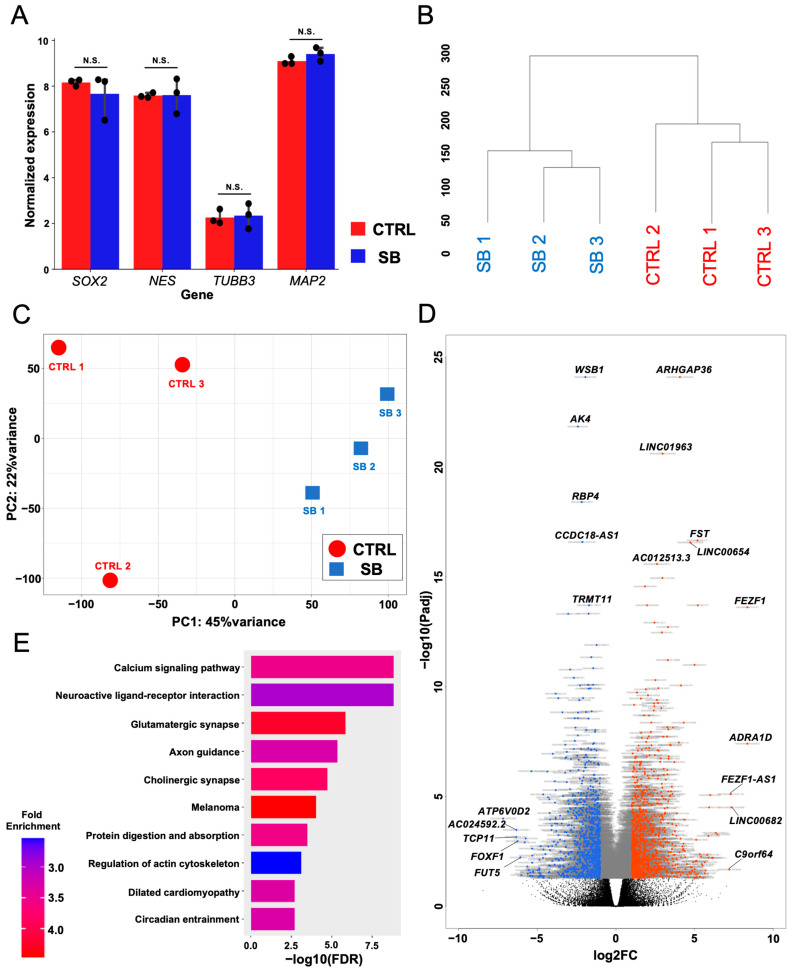
Comprehensive comparison of gene expression levels between the control (CTRL) and sleep bruxism (SB) groups by bulk RNA-seq (CTRL: *n* = 3, SB: *n* = 3). (**A**) Comparison of expression levels of neuronal markers such as *SOX2*, *NES*, *TUBB3*, and *MAP2* between the CTRL and SB groups (N.S. = nonsignificant in Wald test). (**B**) Dendrogram hierarchical clustering analysis for each sample. (**C**) Principal component analysis for each sample. (**D**) Volcano plot from differentially expressed gene (DEG) analysis. The vertical axis is the inverse logarithm of the adjusted *p*-value, and the horizontal axis is the logarithm of the expression ratio of the SB group to the CTRL group. Red dots indicate upregulated genes and blue dots indicate downregulated genes. The top 10 genes with adjusted *p*-values < 0.05 and the top five upregulated and downregulated genes with Log2foldchanges > 1 are shown. (**E**) Top 10 pathways detected by Kyoto Encyclopedia of Genes and Genomes (KEGG) enrichment analysis. The horizontal axis represents the log-transformed false discovery rate (FDR), and the color indicates the fold enrichment value changing from blue to red as the value increases.

The ventral brainstem neurons were differentiated from hiPSCs derived from three patients with SB harboring the C/C genotype of rs6313 (SB1, SB2, and SB3) and three healthy controls harboring the T/T genotype (CTRL1, CTRL2, and CTRL3) based on our previous report [20] (Figure S1). We first applied bulk RNA-seq on these neurons to evaluate changes in comprehensive gene expression patterns in the neurons between the SB and CTRL groups. The expression levels of neuronal markers (*SOX2*, *NES*, *TUBB3*, and *MAP2*) were unchanged between the SB and CTRL groups, indicating comparable differentiation levels between the two groups (Figure 1A). Hierarchical clustering analysis of the transcript per million (TPM) values of all detected gene expressions showed a clear polarization between the CTRL and SB groups, suggesting potential pathophysiological differences between the two groups (Figure 1B). Consistent with the hierarchical clustering analysis, the principal component analysis (PCA) plot demonstrated a distinct separation into the SB and CTRL groups (Figure 1C). These results suggest the presence of SB-specific gene expression patterns.

### 2.2. SB Patient-Specific Gene Expression Profiles of Calcium Signaling Pathways

To clarify the composition of SB-specific gene expression patterns, we subsequently performed a differentially expressed gene (DEG) analysis and identified 2201 DEGs (|Log2FoldChange (Log2FC)| >1, adjusted *p*-value (p_adj_) < 0.05) (Table S1). A total of 1170 genes were significantly upregulated in the SB samples (Log2FC > 1, *p*_adj_ < 0.05), whereas 1031 genes were significantly downregulated (Log2FC < −1, *p*_adj_ < 0.05). These results were visualized using a volcano plot (Figure 1D). Based on the KEGG pathway analysis using 2201 DEGs, the top 10 pathways are listed in Figure 1E. Among these pathways, the enrichment of DEGs in the calcium signaling pathway was most pronounced.

To investigate this pathway, we depicted alterations in gene expression within the calcium signaling pathway on the Pathview map. Alterations in gene expression within this pathway are shown in Figure S2A, and transcripts and their comprising gene groups are listed in Figure S2B. These results revealed changes in the expression of 25 distinct transcript types. The SB samples showed high expression of genes encoding G protein-coupled receptors (GPCRs) and receptor-operated calcium channels (ROCs), which serve as ligands for neurotransmitters. GPCRs and ROCs facilitate signal transduction, a crucial pathway for excitatory neurotransmission between neurons [21,22]. Therefore, subsequent investigations focused on genes encoding these receptors and channels.

**Figure 2:**
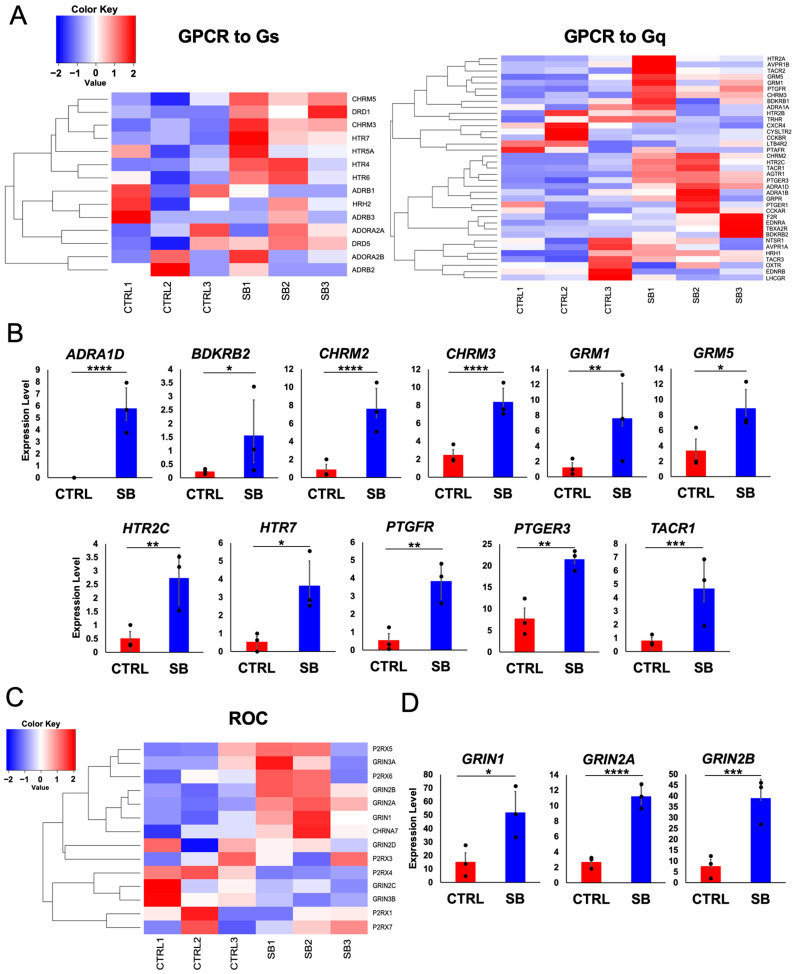
Comparison of the expression levels of genes constituting G protein-coupled receptors (GPCR) and receptor-operated calcium channels (ROC) between the CTRL and SB groups. (**A**) Heatmap visualizing the expression levels of genes constituting GPCRs that activate adenylate cyclase (GPCR to Gs) and phospholipase C (GPCR to Gq) in each sample. Heatmaps were created by Z-scoring the transcript per million (TPM) value of each gene in each sample. (**B**) Graphs of GPCR component genes whose expression levels were significantly upregulated (adjusted *p*-value < 0.05) in the SB samples. The graphs show the mean TPM values for each sample and compare the expression levels of each gene between the CTRL and SB groups (mean ± SE, *n* = 3), * *p*_adj_ < 0.05, ** *p*_adj_ < 0.01, *** *p*_adj_ < 0.001, **** *p*_adj_ < 0.0001. (**C**) Heatmap showing the expression levels of ROC component genes for each sample. This was created using the same method as the GPCR heatmap. (**D**) Graphs of ROC component genes whose expression levels were significantly upregulated (adjusted *p*-value < 0.05) in the SB samples. The graphs show the mean TPM values for each sample and compare the expression levels of each gene between the CTRL and SB groups (mean ± SE, *n* = 3), * *p*_adj_ < 0.05, *** *p*_adj_ < 0.001, **** *p*_adj_ < 0.0001.

The Pathview map illustrates two pathways for GPCRs: one leading to adenylate cyclase activity (GPCR to Gs) and another leading to phospholipase C activity (GPCR to Gq). The heatmap of gene expression for each GPCR pathway showed a tendency toward upregulation of GPCR component genes in the SB samples (Figure 2A). Among these genes, the expression levels of two genes (*CHRM3* and *HTR7*) from the GPCR (to Gs) category and 10 genes (*ADRA1D*, *BDKRB2*, *CHRM2*, *CHRM3*, *GRM1*, *GRM5*, *HTR2C*, *PTGER3*, *PTGFR*, and *TACR1*) from the GPCR (to Gq) category were significantly upregulated in the SB samples compared with the CTRL samples (Figure 2B). Similarly, we visualized the expression levels of genes comprising the ROC pathway in each sample by using a heatmap (Figure 2C) and identified significant upregulation of three genes (*GRIN1*, *GRIN2A*, and *GRIN2B*) in the SB samples (Figure 2D). A comprehensive list of all genes related to the calcium signaling pathway and their TPM values for each sample is provided in the Supplementary Materials (Table S2). Thus, multiple GPCR- and ROC-related genes exhibited elevated expression in the SB group compared with the CTRL group. These results suggest the potential involvement of GPCRs and ROCs, which participate in excitatory transmission, in neuronal hyperactivity in patients with SB.

### 2.3. Analysis of Neuronal Subtype Marker Genes and Identification of Genes Related to the Laterodorsal Tegmental Nucleus

**Figure 3:**
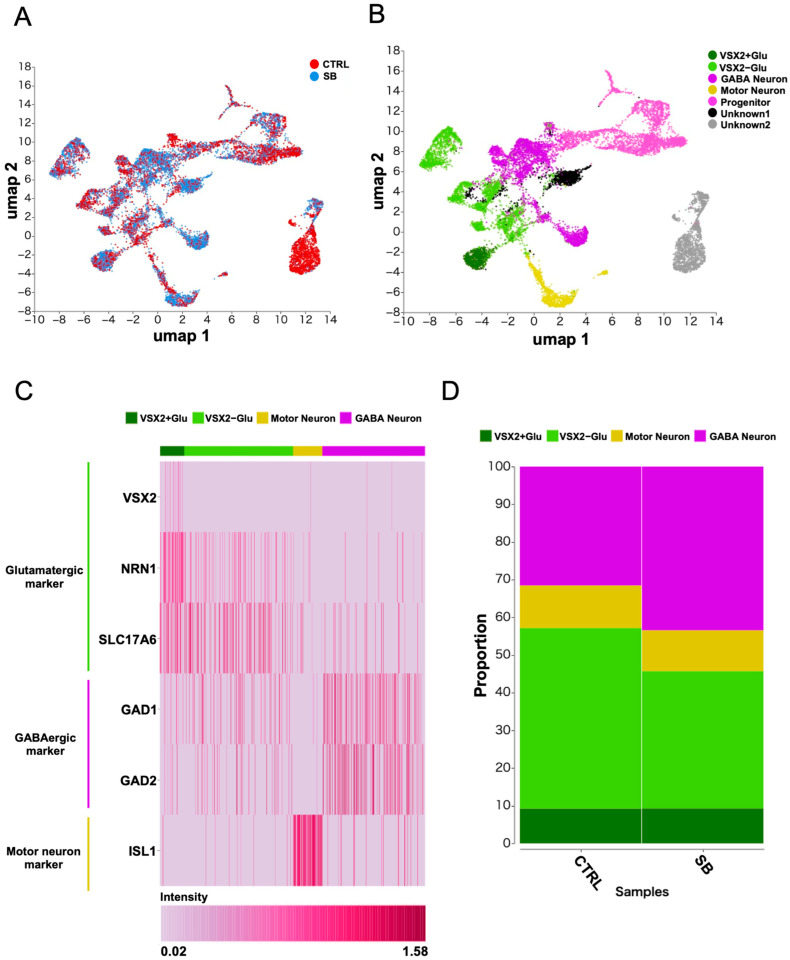
Annotation of neural subtypes in single-nucleus RNA-seq data. (**A**) UMAP embedding of all cells colored by sample origin. *n* = 1. (**B**) UMAP projection showing the subtypes identified by manual annotation. (**C**) Heatmap visualizing the expression of marker genes for the identified neuronal subtypes. (**D**) Histogram depicting the neuronal subtype composition in the CTRL and SB groups.

To identify the pathological cells responsible for SB and detect changes in their gene expression, we performed snRNA-seq on neurons differentiated from hiPSCs derived from a patient with SB (SB2) and a healthy control (CTRL2). The data from CTRL2 and SB2 were integrated, and the gene expression patterns between the samples were visualized using UMAP (Figure 3A). A clustering analysis using the Louvain method identified 16 clusters in the UMAP plot (Figure S3A). To determine the cell subtypes in the clusters, we employed a manual annotation method based on the findings of previous studies [23,24]. Initially, we identified clusters that expressed marker genes for neurons (marker genes: *MAP2*, *SYT1*, *TUBB3*, *ELAVL2*, and *NEFL*) and neural progenitors (marker genes: *NR2F1*, *ZEB2*, *SOX2*, *TOP2A*, and *NES*). Some clusters were unmatched to any of the marker sets and were therefore defined as “Unknown” (Figure S3B). The marker gene expression of each cluster is shown in a heatmap (Figure S3C). Subsequently, marker genes for each neuronal subtype were examined, which resulted in the identification of clusters comprising glutamatergic neurons (marker genes: *SLC17A6* and *NR1*), GABAergic neurons (marker genes: *GAD1* and *GAD2*), and motor neurons (marker gene: *ISL1*). Moreover, certain clusters of glutamatergic neurons exhibited a specific expression of *VSX2*. *VSX2* is selectively expressed when neural progenitor cells differentiate into glutamatergic neurons within the laterodorsal tegmental nucleus (LDT) and pedunculopontine tegmental nucleus (PPT) during development [25,26]. These nuclei are pivotal in inhibiting muscle activity during REM sleep [27]. Considering the possibility that glutamatergic neurons in the differentiated induced neuronal population include those specific to LDT/PPT, we divided the cell population into two clusters: one expressing *VSX2* (VSX2+Glu) and the other lacking its expression (VSX2−Glu). The two neuronal clusters that were challenging to identify using individual marker genes were classified as “Unknown neurons-1” and “Unknown neurons-2”. The UMAP shows the final cluster classification based on the annotation analysis (Figure 3B). The expression patterns of the marker genes were visualized using a heatmap (Figure 3C). A comparison of populations that focused exclusively on neural subtypes revealed no significant difference between the CTRL and SB groups, although the proportion of GABA neurons slightly increased in the SB samples compared with the CTRL samples (Figure 3D).

We performed snRNA-seq and identified neural subtypes, including glutamatergic, GABAergic, and motor neurons. Comparative analysis of the samples revealed that the proportion of neuronal subtypes was marginally different in the SB samples.

### 2.4. Comparison of GRIN2B and CHRM3 Expression Between Neuronal Subtypes in Patients with SB

**Figure 4:**
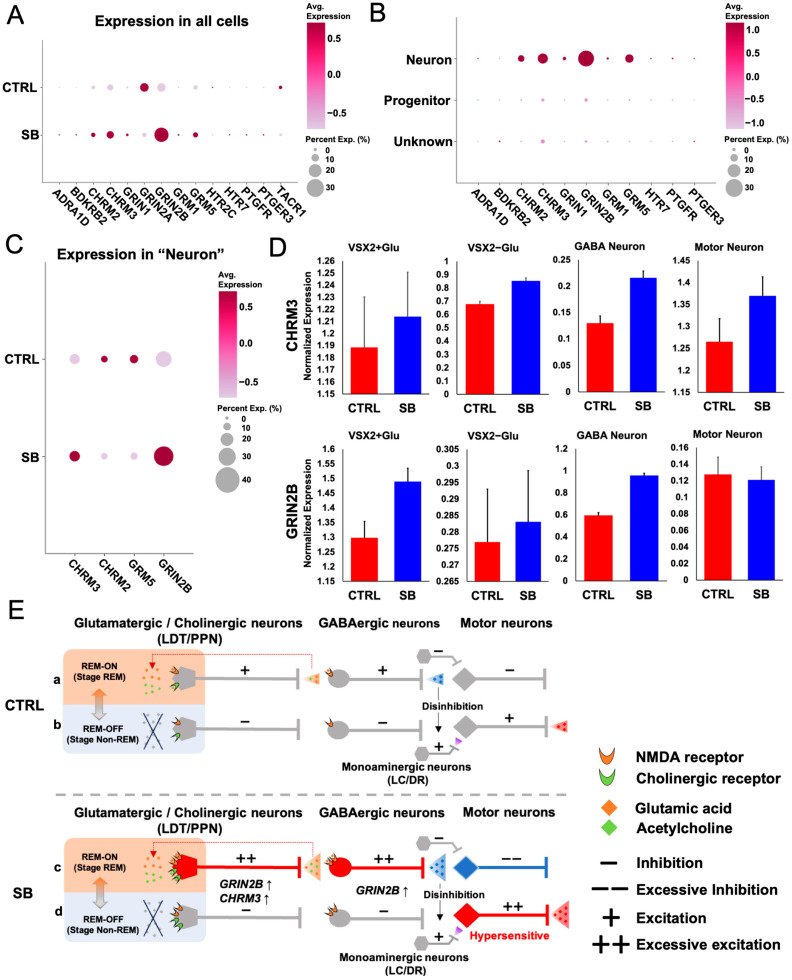
Comparative gene expression analysis in CTRL and SB samples in snRNA-seq data. (**A**) Dot plot showing the expression of 14 genes significantly upregulated in SB samples, as identified by bulk RNA-seq within overall cells. Dot size represents the percentage of cells expressing each gene, while color intensity indicates the average scaled expression level. (**B**) Dot plot displaying the expression levels of 11 genes that were increased in the SB samples, as presented in Figure 4A, across different cell types. (**C**) Dot plot comparing the expression of cholinergic receptor genes (*CHRM2* and *CHRM3*) and glutamatergic receptor genes (*GRIN2B* and *GRM5*) between the CTRL and SB samples within the neuronal subtype. (**D**) Graphs depicting the expression values of *CHRM3* and *GRIN2B* across different neuronal subtypes. Bars represent the mean expression of each cell, with the standard error indicated by the error bars. (**E**) Summary chart of the hypothesized mechanisms of SB pathogenesis suggested by the results of this study.

To verify whether the expression trends of the 14 genes with significantly upregulated expression in the SB samples from bulk RNA-seq data are preserved, we conducted a comparative analysis using snRNA-seq data. Eleven genes (*ADRA1D*, *BDKRB2*, *CHRM2*, *CHRM3*, *GRIN1*, *GRIN2B*, *GRM1*, *GRM5*, *HTR7*, *PTGFR*, and *PTGER3*) were upregulated in SB, generally preserving the trends observed in the bulk RNA-seq (Figure 4A). To identify genes associated with neuronal responsiveness, we visualized the upregulated genes in the SB group across each cell subtype. The expression levels of acetylcholine receptor-related genes (*CHRM2* and *CHRM3*) and glutamate receptor-related genes (*GRIN2B* and *GRM5*) were enriched in the neuronal subtypes compared with the non-neuronal subtypes (Figure 4B). Additionally, within neuronal cells, the expression levels of *CHRM2* and *GRM5* were downregulated in the SB group (Figure 4C). Based on these results, *CHRM3* and *GRIN2B* were selected for further examination. These results indicated that the expression of the candidate genes was significantly elevated in specific neuronal subtypes in the SB samples.

We compared gene expression in each neuronal subtype in the CTRL and SB samples to identify the pathological cells responsible for SB. The expression of *CHRM3* was upregulated in all neuronal subtypes, whereas that of *GRIN2B* was upregulated specifically in glutamatergic and GABAergic neurons (Figure 4D). These findings suggested that enhanced expression of these genes contributed to excessive neuronal excitation in the SB samples.

## 3. Discussion

In this study, we conducted a comprehensive gene expression analysis using neurons derived from three patients with SB to investigate the pathophysiology of SB. Bulk RNA-seq revealed significant systematic differences between the SB and CTRL groups, including an upregulation of genes related to calcium signaling pathways. In particular, the expression of specific receptors, such as N-methyl-D-aspartate (NMDA) and acetylcholine receptors, was significantly elevated in SB. Furthermore, the snRNA-seq analysis demonstrated increased expression of *GRIN2B* and *CHRM3* in glutamatergic and GABAergic neurons and *CHRM3* expression in motor neurons derived from patients with SB.

### 3.1. Calcium Signaling Dysregulation as a Potential Mechanism for Neuronal Activity Imbalance in SB

Calcium signaling plays a critical role as an intracellular messenger in releasing neurotransmitters and excitation transmission [28,29,30]. Disruptions in calcium signaling have been implicated in imbalances of neural activity, contributing to conditions such as epilepsy and sleep disorders [31,32]. Although no studies have directly implicated calcium signaling in SB pathology, our previous research using the whole-cell patch-clamp method demonstrated that neurons derived from patients with SB exhibit increased firing frequency and gain and shorter half-life in action potential [20]. These results suggest that aberrant calcium signaling contributes to neuronal hyperexcitability in SB. Calcium imaging can be employed in the future to visualize calcium signaling dynamics in neurons derived from patients with SB, enabling a detailed exploration of these mechanisms.

### 3.2. GRIN2B and CHRM3 Overexpression as Drivers of Neuronal Hyperexcitability in SB

*GRIN2B* encodes the NR2B subunit of the NMDA receptor and plays an essential role in excitatory signal transduction in neurons [33]. Overexpression of *GRIN2B* results in NMDA receptor hyperactivation and serves as a risk factor for epilepsy [34,35]. Additionally, research using mouse models of neurodevelopmental disorders has shown that *GRIN2B* mutations can manifest in autism spectrum disorders and intellectual disabilities [36] and are often accompanied by abnormal sleep behaviors, including SB [37,38]. These findings suggest that the elevated expression of *GRIN2B* in neurons of patients with SB drives abnormal sleep behaviors through neuronal hyperexcitability.

*CHRM3* encodes the M3 muscarinic acetylcholine receptor, a GPCR involved in signal transduction, by binding to the neurotransmitter acetylcholine [39,40]. Previous studies have suggested that *CHRM3* knockout mice exhibit reduced non-REM sleep stages [41]. However, the impact of *CHRM3* overexpression on sleep has not been examined. Given that no differences in sleep phases have been observed between patients with SB and healthy individuals [10], the elevated expression of *CHRM3* in neurons from patients with SB may be associated with a mechanism distinct from sleep. Notably, *CHRM3* is highly expressed in motor neurons, suggesting that it may directly contribute to increased muscle activity in patients with SB.

### 3.3. Impact of SNP rs6313 on GRIN2B and CHRM3 Expression in SB Neurons

The increased expression of *GRIN2B* and *CHRM3* in neurons from patients with SB may be attributed to the effects of SNPs. The SB samples used in this study were hiPSC-derived neurons from three patients with SB (C/C genotype), all of which carried the C allele at SNP rs6313 in *HTR2A*, which has been previously reported as a risk factor for SB [18,19,20]. rs6313 is primarily associated with *HTR2A* expression and the function of the 5-HT2A receptor [42]. Notably, C allele carriers show increased mRNA levels of *HTR2A*, potentially causing hyperactivity of the 5-HT2A receptor and excessive signal activation [43]. Although no direct association between the muscarinic receptor and the 5-HT2A receptor has been established, a connection between rs6313 and NMDA receptors has been reported. A previous study demonstrated that 5-HT2A receptor activation enhances NMDA receptor function via Src kinase, amplifying glutamate responses [44]. This mechanism suggests that rs6313 polymorphisms alter serotonin signaling, increasing neural activity mediated by NMDA receptors. This may lead to elevated expression of *GRIN2B*, particularly in glutamatergic and GABAergic neurons.

No studies have reported an association between rs6313 and *GRIN2B* or *CHRM3*. Other SNPs and copy number variants possibly contributed to the SB-specific gene expression changes identified in this study. Therefore, comprehensive genetic analyses, including whole-genome and exome sequencing, are essential. This approach is expected to elucidate further genetic factors associated with SB.

### 3.4. Hypothesis on the Mechanisms Underlying SB-Related Muscle Activity

Muscle atonia during REM sleep is primarily attributed to motor neuron inhibition mediated by glutamatergic and cholinergic neurons in the LDT and PPT via GABAergic interneurons [27,45]. These nuclei integrate inputs from the cerebral cortex and hypothalamus and maintain reciprocal connections [46]. The release of glutamate and acetylcholine in the LDT and PPT induces self-activation of these nuclei, which is crucial for increased neuronal activity within these regions and thereby regulates REM sleep-related atonia. Moreover, the locus coeruleus (LC) and dorsal raphe nucleus (DR) play a significant role in this process. During REM sleep, the activity of monoaminergic neurons from the LC and DR to motor neurons decreases. This decline in excitatory input to the motor neurons further suppresses motor activity, thereby enhancing muscle atonia during REM sleep [47] (Figure 4E(a)).

The inhibition of motor neurons during REM sleep is alleviated during non-REM sleep. Body movements are relatively frequent in stages N1 and N2 of non-REM sleep, whereas muscle tone is still present in N3 [48,49]. This phenomenon arises from the decreased activity of glutamatergic and cholinergic neurons, including those located in the LDT and PPT, resulting in diminished GABAergic inhibition. The reduction in inhibitory input facilitates the excitation of motor neurons, thereby sustaining muscle tone during non-REM sleep [50]. Moreover, excitatory input from monoaminergic neurons to motor neurons is maintained at moderate levels, although less than during wakefulness. This sustained excitatory drive allows motor neurons to shift into an active state, contributing to preserving motor function during sleep (Figure 4E(b)).

In the present study, *VSX2*-positive cells in the differentiated neurons indicated that neurons in the LDT/PPT region were induced. Furthermore, the VSX2+Glu cluster contained *CHAT*-positive cells, suggesting cholinergic characteristics in some neurons (Figure S4). *GRIN2B* and *CHRM3* expression was upregulated in the VSX2+Glu cluster of the SB samples, whereas *GRIN2B* expression was upregulated in the GAD1+/GAD2+ GABA clusters. This result suggests the potential expression of *CHRM3* and *GRIN2B* in glutamatergic/cholinergic and GABAergic neurons within the motor neuron inhibitory pathway, enhancing acetylcholine and NMDA receptor functionality. Based on these findings, we propose the following hypothesis regarding the mechanism of motor neuron hyperactivity that causes SB. In patients with SB, glutamatergic/cholinergic neurons in the LDT/PPT region may enhance the activity of GABAergic neurons, resulting in stronger inhibition of motor neuron activity during REM sleep compared with CTRL (Figure 4E(c)). The phenomenon by which neurons exhibit enhanced firing activity after being released from inhibition is known as rebound firing [51]. In patients with SB, removal of strong GABAergic inhibition during REM sleep has been hypothesized to lead to hyperresponsive motor neurons, resulting in increased sensitivity to the same excitatory input compared with CTRL. This phenomenon may lead to excessive motor responses (Figure 4E(d)).

Excitatory input from monoaminergic neurons in the LC and DR, which sustains muscle activity during non-REM sleep, may increase with sympathetic nervous system activity [52,53,54]. Patients with SB display RMMA, which is accompanied by activation of the sympathetic nervous system, whereas healthy controls exhibit no SB symptoms despite the presence of MA [13,14]. These findings indirectly support the hypothesis.

To verify this hypothesis, we must investigate the strong inhibitory effect in neurons from patients with SB and the roles of *CHRM3* and *GRIN2B*. Future studies will focus on the electrophysiological characteristics of glutamatergic/cholinergic, GABAergic, and motor neuron subtypes, which will be separately cultured and isolated from differentiated cell populations. Furthermore, phenotypic alterations resulting from the knockdown of *CHRM3* and *GRIN2B* will be examined. Additionally, efforts will be directed toward developing SB disease animal models incorporating the proposed neural circuit.

If this hypothesis is validated, targeting *GRIN2B*, which may play a crucial role in the excessive suppression of SB, could provide a viable therapeutic approach. One promising candidate is memantine, an NMDA receptor antagonist that is currently approved for Alzheimer’s disease. Notably, previous studies reported that memantine inhibits periodic limb movements during sleep, which are associated with SB [55] in patients with Alzheimer’s disease [56]. By inhibiting excessive NMDA receptor activation during REM sleep, memantine may attenuate the over-suppression of motor neurons and consequently prevent abnormal motor neuron responses during disinhibition.

### 3.5. Limitations

Despite providing valuable insights into the molecular mechanisms underlying SB, this study has several limitations. First, the sample size was relatively small, with only three patient-derived and three control hiPSC lines. This limited sample size may affect the generalizability of the findings, as individual variability could influence the gene expression patterns in neurons derived from patients with SB. Additionally, although hiPSCs provide a relevant model for studying patient-specific neuronal characteristics, the in vitro environment cannot fully recapitulate the complex interactions and environmental factors of the human brain in vivo. Furthermore, the study focused primarily on bulk and single-nucleus RNA-seq, which may not capture other levels of regulation, such as protein expression or post-translational modifications, which also play crucial roles in neuronal hyperexcitability. The reliance on hiPSC-derived brainstem neurons, while valuable for understanding SB-specific excitability, does not account for potential contributions from other neural circuits or systemic factors involved in the SB. Future studies with larger sample sizes, in vivo models, and multiomic approaches are essential to comprehensively understand the pathophysiology of SB.

## 4. Materials and Methods

### 4.1. Ethics Statement

The following protocol was approved by the Showa University Ethics Committee for Genome Research (approval no. 179) and the Juntendo University School of Medicine Ethics Committee (approval no. 2016117). The study protocol adhered to the 1964 Declaration of Helsinki and its later amendments or comparable ethical standards. Formal informed consent was obtained from each participant.

### 4.2. iPSC Culture

The hiPSCs of three patients with SB (SB1, SB2, and SB3), all harboring the risk allele (C) of HTR2A SNP rs6313, and three unaffected controls (CTRL1, CTRL2, and CTRL3) without the risk allele were used. The patients with SB were selected based on modified clinical diagnostic criteria for SB: (1) reports of tooth-grinding sounds by their sleep partner; (2) presence of tooth attrition with exposed dentin; (3) reports of morning masticatory muscle fatigue or tenderness; or (4) presence of masseter muscle hypertrophy [17]. Furthermore, the frequency of SB episodes per hour of sleep was determined using masseter electromyographic data recorded during sleep using polysomnography. Detailed data on genotypes and clinical characteristics of the groups are presented in Table S3.

The hiPSC lines from the patients (SB1, SB2, and SB3) and controls (CTRL1, CTRL2, and CTRL3) were previously established [19,20]. All hiPSC lines were cultured on plates coated with iMatrix-511 (Nippi, Shizuoka, Japan) and expanded in StemFit AK02N medium (Ajinomoto, Tokyo, Japan) in an atmosphere containing 3% CO_2_. The medium was changed every other day. For this study, the passage numbers of the hiPSC lines were carefully controlled to minimize variability due to differences in cellular age or genetic stability. The passage numbers were as follows: CTRL1, 45 passages; CTRL2, 40 passages; CTRL3, 34 passages; SB1, 44 passages; SB2, 45 passages; and SB3, 20 passages.

### 4.3. Neuronal Differentiation

Neuronal differentiations were performed using the previously portrayed paradigm with slight modifications (Figure S1A). We generated neurons with the characteristics of the brainstem region [19,20]. The hiPSCs were pretreated for 5 days with 3 μM SB431542 (Tocris, Bristol, UK), 3 μM dorsomorphin (Sigma-Aldrich, St. Louis, MO, USA), and 3 μM CHIR99021 (Stemgent, Cambridge, MA, USA). Chemically transitional embryoid-body-like cells called CTraS [57] were seeded at a density of 10 cells/μL in KBM Neural Stem Cell medium (Kohjin-Bio, Saitama, Japan) with specifically selected growth factors and inhibitors at 4% O2 and 5% CO_2_; growth factors and inhibitors included 1 × B-27 supplement (Gibco; Thermo Fisher Scientific, Waltham, MA, USA), 0.5% penicillin/streptomycin, 20 ng/mL FGF-2, 2 μM SB431542, and 3 μM CHIR99021. The neurosphere culture was started at zero days in vitro (DIV); neurospheres were passaged at a density of 50 cells/μL on DIV7 and DIV14. The neurosphere culture medium contained the following additives: 10 μM Y-27632 (Fujifilm, Tokyo, Japan) on DIV0–7, and 100 ng/mL sonic hedgehog (Shh-C24II; R&D Systems, Minneapolis, MN, USA) and 1 μM purmorphamine (Millipore, Burlington, MA, USA) on DIV1–14. On DIV14, neurospheres were replated on dishes coated with poly-L-lysine (Sigma-Aldrich) and fibronectin (Corning, Corning, NY, USA) and cultured in 5% CO_2_ at a density of 2 × 10^6^ cells/well in a 6-well plate. The medium was replaced with Neurobasal Plus medium (Thermo Fisher Scientific, Waltham, MA, USA) supplemented with 1 × B-27 Plus supplement, 0.5% penicillin/streptomycin, 20 ng/mL brain-derived neurotrophic factor (BDNF; BioLegend, San Diego, CA, USA), 20 ng/mL glial cell-derived neurotrophic factor (GDNF; Alomone Labs, Jerusalem BioPark, Jerusalem, Israel), 0.2 mM ascorbic acid (Sigma-Aldrich), 0.5 mM dbcAMP (Nacalai Tesque, Kyoto, Japan), 1 ng/mL transforming growth factor-β (TGF-β; BioLegend), 10 μM DAPT (Sigma-Aldrich), and 3 μM CHIR99021. Half of the medium volume was replaced with fresh medium (including all supplements except CHIR99021) every 3 or 4 days. The timing and duration of each stage were optimized based on a combination of previously published protocols [19,20,57] and preliminary experiments in our laboratory. For example, the incubation period during neurosphere formation (DIV0–14) was determined to allow optimal aggregation and neurosphere proliferation. Similarly, neurosphere density during passaging (50 cells/μL) on DIV7 and DIV14 was selected to minimize cellular stress and promote consistent growth.

### 4.4. Bulk RNA-Seq

Neurons (2 well/6-well plate; 2 × 10^6^ cells/well for each sample) were induced to differentiate from three samples of hiPSCs derived from patients with SB (SB1, SB2, and SB3) and three healthy controls (CTRL1, CTRL2, and CTRL3) (Figure S1B). Neurons were collected at DIV35 based on previous studies demonstrating neuronal maturation between DIV31 and DIV51 [20]. Preliminary experiments showed that cultures beyond DIV35 resulted in significant neuronal aggregation and increased cell death. Total RNA was extracted from DIV35 neurons using TRIZOL Reagent (Thermo Fisher Scientific) following the manufacturer’s protocol. The extracted RNA was further purified and enriched using NucleoSpin RNA Clean-up XS (Macherey-Nagel, Düren, Germany) in accordance with the manufacturer’s protocol. Each RNA sample was evaluated for purity and concentration using BioDrop µLite (BioDrop, Cambridge, UK). The A260:A280 ratio was 1.8–2.2, the A260:A230 ratio was 1.8 or higher, and the concentration was 50 ng/µL. Sequences of each sample were obtained after library preparation by the poly-A selection method using NovaSeq 6000 (Illumina, San Diego, CA, USA).

Quality control measurements were performed using FastQC v0.11.7 and Trimmomatic v.0.38. Sequence reads were trimmed and mapped to the human reference genome (GRCh38) using the HISAT2 v2.1.0 [58] alignment tool. BAM files were prepared using SAMtools v1.11 [59]. The number of raw reads mapped to known exon regions was calculated using featureCounts v1.6.3 [60]. Raw read counts were normalized to TPM. The samples were clustered using the Wald method based on Euclidean distances.

DEGs were normalized using relative log normalization and identified using DESeq2 v1.24.0. DEGs with an absolute value of log fold change (log2FC) ≥ 1 and adjusted *p*-value < 0.05 by the Benjamini and Hochberg method were selected. KEGG pathway analysis was performed to classify the DEGs into functional protein classes using the enrichKEGG function in the ClusterProfiler [61] package. Gene expression data were projected onto pathway maps of the top hits using Pathview (https://pathview.uncc.edu accessed on 16 January 2024).

### 4.5. Single-Nucleus RNA-Seq

Neurons (2 wells per 6-well plate; 2 × 106 cells/well for each sample) were induced to differentiate from one sample of hiPSC (SB2) and one control sample (CTRL2) derived from patients with SB (Figure S1B). Following the protocol for bulk RNA-seq, Div35 neurons were harvested using Accutase (Thermo Fisher Scientific) and processed using the 10x Genomics protocol for nuclei isolation (Nuclei Isolation for Single Cell Multiome ATAC + Gene Expression). Naked nucleus manipulation was performed in accordance with the Chromium Next GEM Single Cell 3′ Reagent Kits v3.1 (Dual Index; Illumina) CG 000,315 Rev E protocol. This was followed by cDNA synthesis and the preparation of gene expression libraries. The quality of the cDNA and libraries was confirmed by electrophoresis.

UMI quantification was performed using Cell Ranger (version 7.1.0) with default parameters, generating a matrix of UMI counts per cell. The single-nucleus RNA-seq dataset was processed, analyzed, and visualized using Trailmaker™ (https://app.trailmaker.parsebiosciences.com accessed on 5 August 2024) hosted by Parsebioscience. A pre-filtered count matrix was uploaded, and additional filtering was performed. Barcodes with mitochondrial reads exceeding 15.87% in CTRL2 and 12.53% in SB2 and barcodes with a doublet score higher than 0.5 were removed. After filtering, 6856 high-quality barcodes were retained for CTRL2 and 7311 for SB2. Data were log-normalized, and the top 2000 highly variable genes were selected using variance-stabilizing transformation. PCA was performed with the top 35 principal components, explaining 91.34% of the total variance. These components were batch-corrected using the Harmony package in R. Clustering was performed using Seurat’s Louvain method with the resolution set to 0.7, and the results were visualized in a UMAP plot. All clusters were manually annotated based on the existing literature. Gene expression heatmaps, dot plots, and violin plots were generated using Trailmaker™.

### 4.6. Statistical Analysis

All data analyses were conducted using the R programming language (version 4.3.2), supplemented with visualization and additional analysis tools Trailmaker™ (Parse Biosciences) and Excel (Microsoft Office, version 16.78). Comparison in bulk RNA-seq analysis was performed using the Wald test, with adjusted *p*-values less than 0.05 considered statistically significant.

## 5. Conclusions

We performed bulk and single-nucleus RNA sequencing on neurons differentiated from hiPSCs derived from patients with SB. We identified alterations in the expression of genes associated with calcium signaling and increases in the expression of *CHRM3* and *GRIN2B* in specific neuronal subtypes. These findings suggest that disruptions in calcium signaling pathways and crucial genes such as *CHRM3* and *GRIN2B* play pivotal roles in the neurophysiological mechanisms underlying SB-related muscle activity. This study is the first to conduct a comprehensive gene expression analysis on neurons derived from patients with SB. Understanding gene expression changes is essential to elucidate the pathophysiology of SB, and our results provide foundational insights that could facilitate the development of disease models and novel therapeutic approaches in the future.

## Data Availability

All the transcriptome raw data that we sequenced were deposited in the NCBI gene expression omnibus (GEO; accession numbers, GSE277985 and GSE278284).

## Supplementary Materials

The following supporting information can be downloaded at: https://www.mdpi.com/article/10.3390/ijms252313141/s1. References [57,62] are cited in the Supplementary Materials.
