# Supplementary material for: Comprehensive Gene Expression Analysis Using Human Induced Pluripotent Stem Cells Derived from Patients with Sleep Bruxism: A Preliminary In Vitro Study
Source: Int J Mol Sci. 2024 Dec 6;25(23):13141. doi: 10.3390/ijms252313141 (PMC11642595; doi:10.3390/ijms252313141)
Supplement: Supplementary file 1 [file ijms-25-13141-s001.zip › Supplementary figure.pdf]

Figure S1

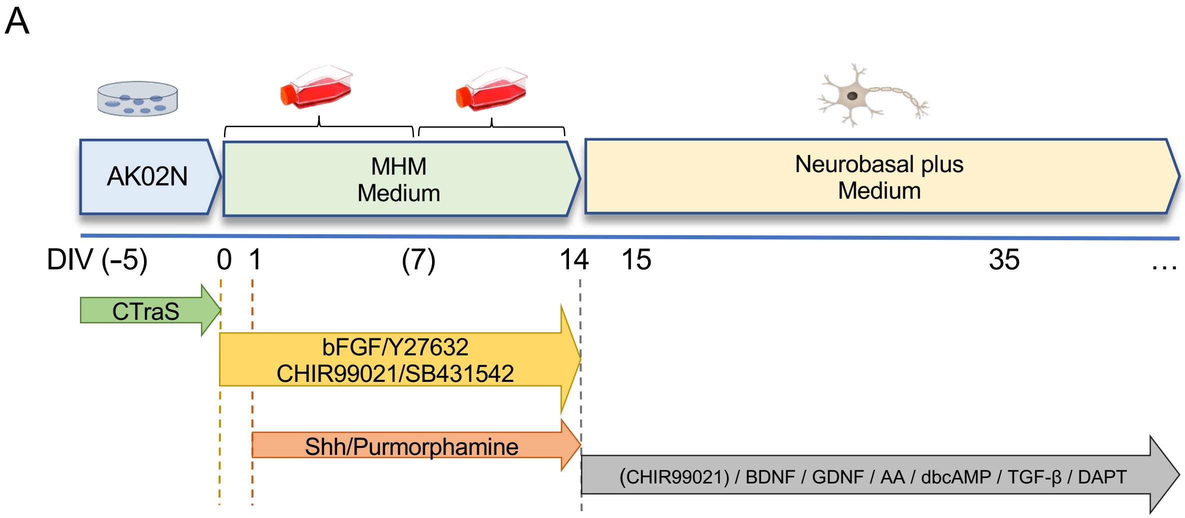
A
AK02N
MHM
Medium
Neurobasal plus
Medium
DIV (-5)
0
1
(7)
14
15
35
...
CTraS
bFGF/Y27632
CHIR99021/SB431542
Shh/Purmorphamine
(CHIR99021) / BDNF / GDNF / AA / dbcAMP / TGF-β / DAPT

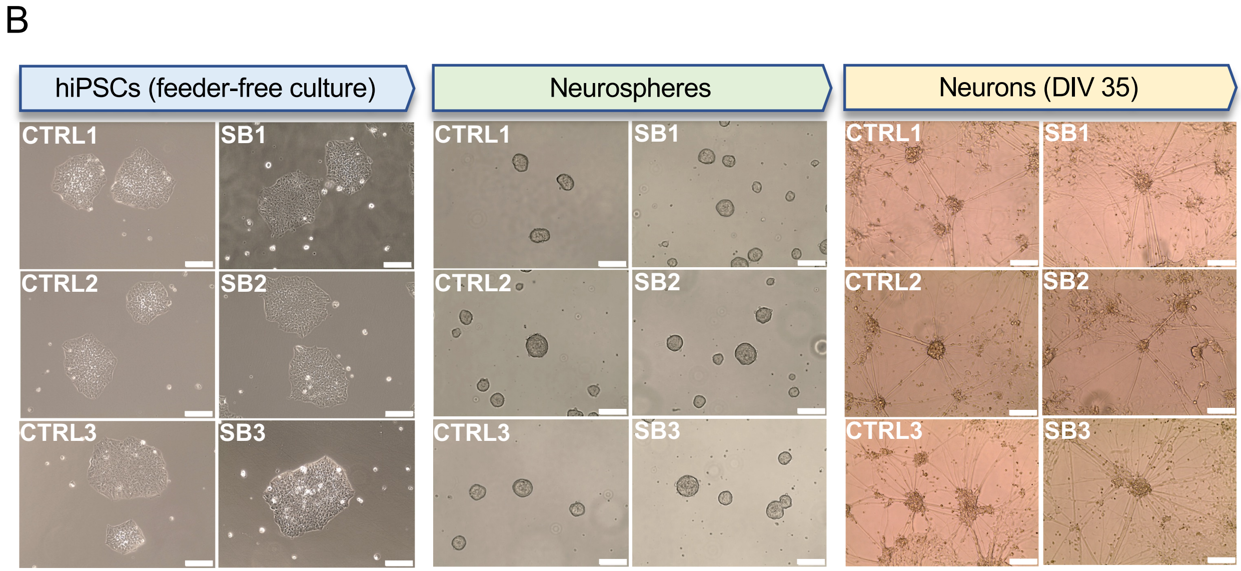
B
hiPSCs (feeder-free culture)
Neurospheres
Neurons (DIV 35)
CTRL1
SB1
CTRL2
SB2
CTRL3
SB3
CTRL1
SB1
CTRL2
SB2
CTRL3
SB3
CTRL1
SB1
CTRL2
SB2
CTRL3
SB3

Figure S2

A

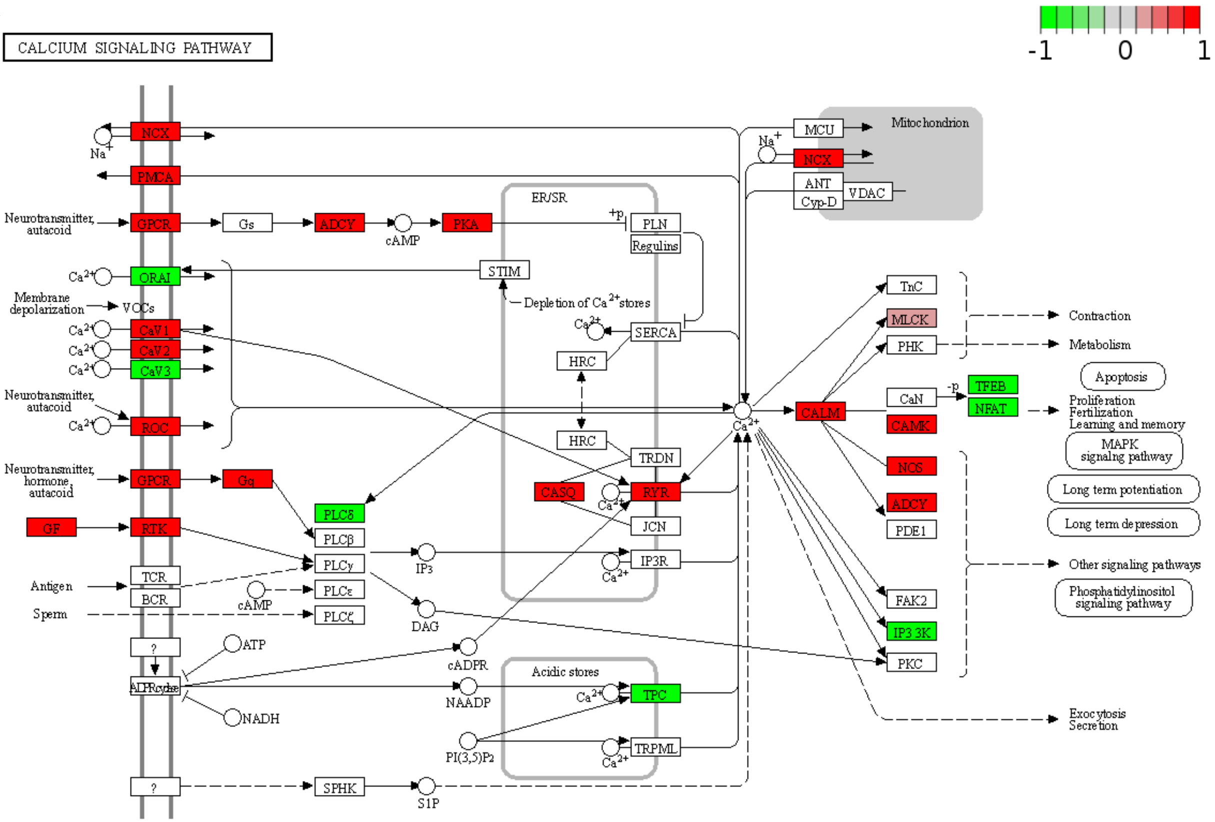

B

Upregulated (adj. p-val. < 0.05)

	GeneID	gene_sym	log2FoldChange(SB/CTRL)	pvalue	padj
**NCT**	ENSG00000118160	SLC8A2	1.544454296	6.07E-07	5.30E-05
	ENSG00000100678	SLC8A3	1.2388008	0.00071793	0.01201434
**PMCA**	ENSG00000157087	ATP2B2	1.60907451	4.36E-05	0.00141332
	ENSG00000067842	ATP2B3	1.25247096	0.0006381	0.01099709
**GPCR (to Gs)**	ENSG00000133019	CHRM3	1.733336701	4.20E-07	4.00E-05
	ENSG00000148680	HTR7	2.710475724	0.0009255	0.01464574
**CaV1**	ENSG00000151067	CACNA1C	1.207383052	1.82E-06	0.00012124
**CaV2**	ENSG00000148408	CACNA1B	1.301120745	6.15E-09	1.30E-06
	ENSG00000198216	CACNA1E	1.356613565	0.00021927	0.00487917
**ROC**	ENSG00000176884	GRIN1	1.764837004	0.00075464	0.01251912
	ENSG00000183454	GRIN2A	2.035290774	2.63E-13	2.45E-10
	ENSG00000273079	GRIN2B	2.350503835	8.35E-06	0.00040071
**GPCR (to Gq)**	ENSG00000171873	ADRA1D	8.350147697	8.58E-11	3.85E-08
	ENSG00000168398	BDKRB2	2.70439596	0.00219127	0.02775413
	ENSG00000181072	CHRM2	3.116382666	7.25E-07	6.05E-05
	ENSG00000133019	CHRM3	1.733336701	4.20E-07	4.00E-05
	ENSG00000152822	GRM1	2.618810596	0.00016738	0.00400628
	ENSG00000168959	GRM5	1.398929793	0.00396524	0.04324823
	ENSG00000147246	HTR2C	2.412508219	0.0001842	0.0042847
	ENSG00000050628	PTGER3	1.47022501	6.17E-05	0.0018517
	ENSG00000122420	PTGFR	2.823128405	0.00012949	0.00329661
	ENSG00000115353	TACR1	2.537523057	1.07E-05	0.00049193

	GeneID	gene_sym	log2FoldChange(SB/CTRL)	pvalue	padj
**Gq**	ENSG00000088256	GNA11	1.06373584	3.02E-10	1.08E-07
	ENSG00000115353	TACR1	2.537523057	1.07E-05	0.00049193
**GF**	ENSG00000168621	GDNF	2.873198148	0.00011913	0.00309584
	ENSG00000102678	FGF9	1.239056172	0.00140766	0.01997356
	ENSG00000070193	FGF10	3.167331678	0.000326	0.00659965
	ENSG00000156427	FGF18	1.007181293	0.00102293	0.01576297
	ENSG00000162344	FGF19	3.616117264	0.00234101	0.02898852
	ENSG00000170962	PDGFD	3.458356188	5.95E-08	8.45E-06
**RTK**	ENSG00000105976	MET	2.294171196	5.63E-06	0.00029843
	ENSG00000140538	NTRK3	1.174373927	5.89E-05	0.00178535
	ENSG00000134853	PDGFRA	2.530635515	1.26E-05	0.00054975
**ADCY**	ENSG00000164742	ADCY1	1.229758436	0.00022111	0.00491705
**RYR**	ENSG00000198626	RYR2	2.280403397	4.41E-05	0.00142433
	ENSG00000198838	RYR3	1.824761503	2.97E-06	0.00018006
**CASQ**	ENSG00000118729	CASQ2	4.048112666	0.00204022	0.02631173
**NCX**	ENSG00000118160	SLC8A2	1.544454296	6.07E-07	5.30E-05
	ENSG00000100678	SLC8A3	1.2388008	0.00071793	0.01201434
**MLCK**	ENSG00000145949	MYLK4	2.533003075	0.00054603	0.00976647
**CAMK**	ENSG00000058404	CAMK2B	1.517311061	3.42E-07	3.40E-05
**NOS**	ENSG00000089250	NOS1	3.353117056	4.43E-10	1.46E-07
**ADCY**	ENSG00000164742	ADCY1	1.229758436	0.00022111	0.00491705

Downregulated (adj. p-val. < 0.05)

	GeneID	gene_sym	log2FoldChange(SB/CTRL)	pvalue	padj
**ORAI**	ENSG00000175938	ORAI3	-1.348330628	0.00019526	0.00445929
**CaV3**	ENSG00000006283	CACNA1G	-1.57845939	3.20E-07	3.24E-05
**PLC δ**	ENSG00000187091	PLCD1	-2.4193168	4.15E-12	2.65E-09
	ENSG00000115556	PLCD4	-2.156266	0.0001717	0.00406717

	GeneID	gene_sym	log2FoldChange(SB/CTRL)	pvalue	padj
**TPC**	ENSG00000186815	TPCN1	-1.509828989	2.16E-06	0.00013857
**IP3 3K**	ENSG00000143772	ITPKB	-2.16654164	1.31E-08	2.49E-06
**TFEB**	ENSG00000112561	TFEB	-1.922997808	0.00430495	0.04593075
**NFAT**	ENSG00000100968	NFATC4	-1.033757363	0.00435438	0.04628608

Figure S3

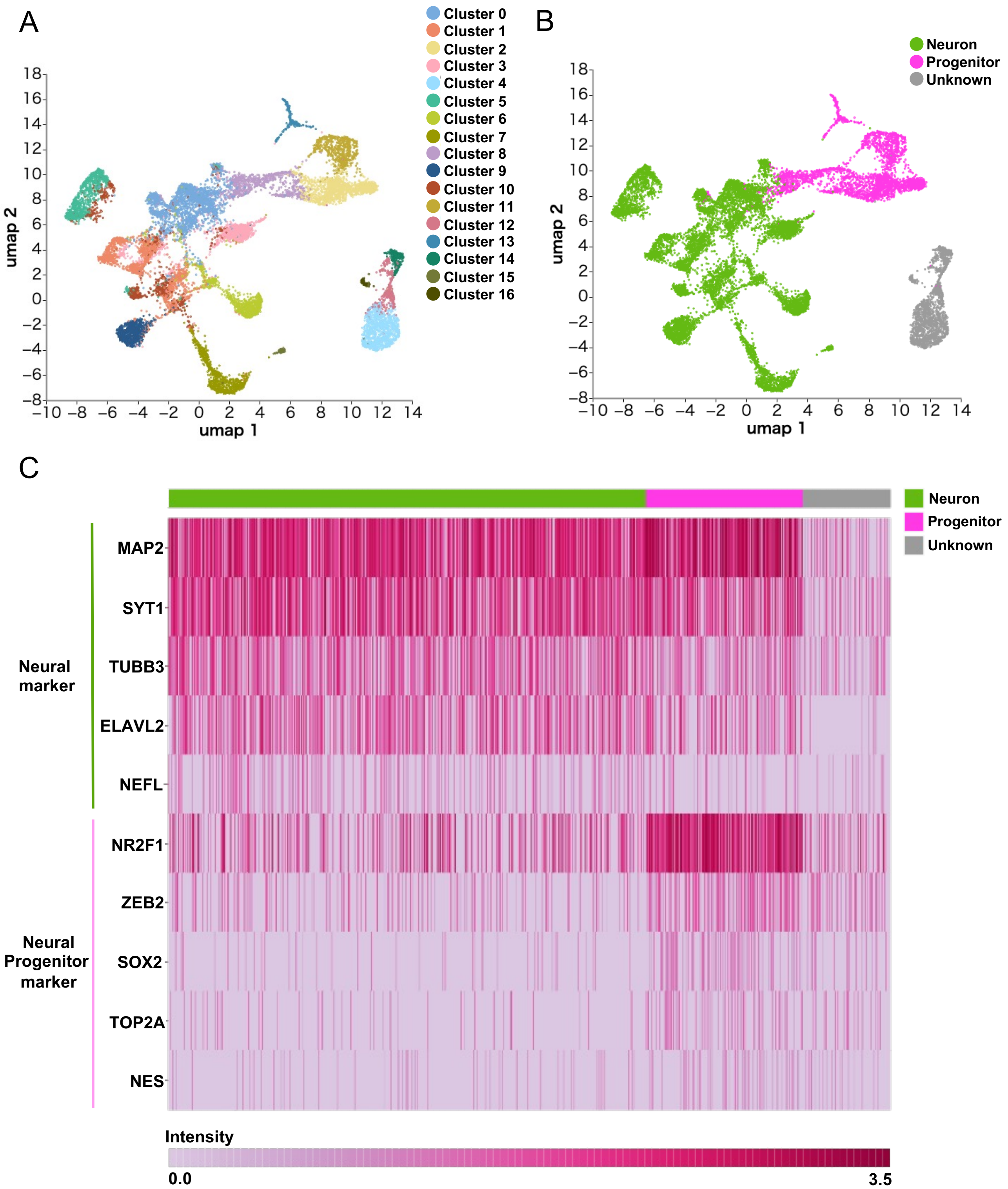
A
B
C
Cluster 0
Cluster 1
Cluster 2
Cluster 3
Cluster 4
Cluster 5
Cluster 6
Cluster 7
Cluster 8
Cluster 9
Cluster 10
Cluster 11
Cluster 12
Cluster 13
Cluster 14
Cluster 15
Cluster 16
umap 1
umap 2
Neuron
Progenitor
Unknown
Neural marker
MAP2
SYT1
TUBB3
ELAVL2
NEFL
Neural Progenitor marker
NR2F1
ZEB2
SOX2
TOP2A
NES
Intensity
0.0
3.5

Figure S4

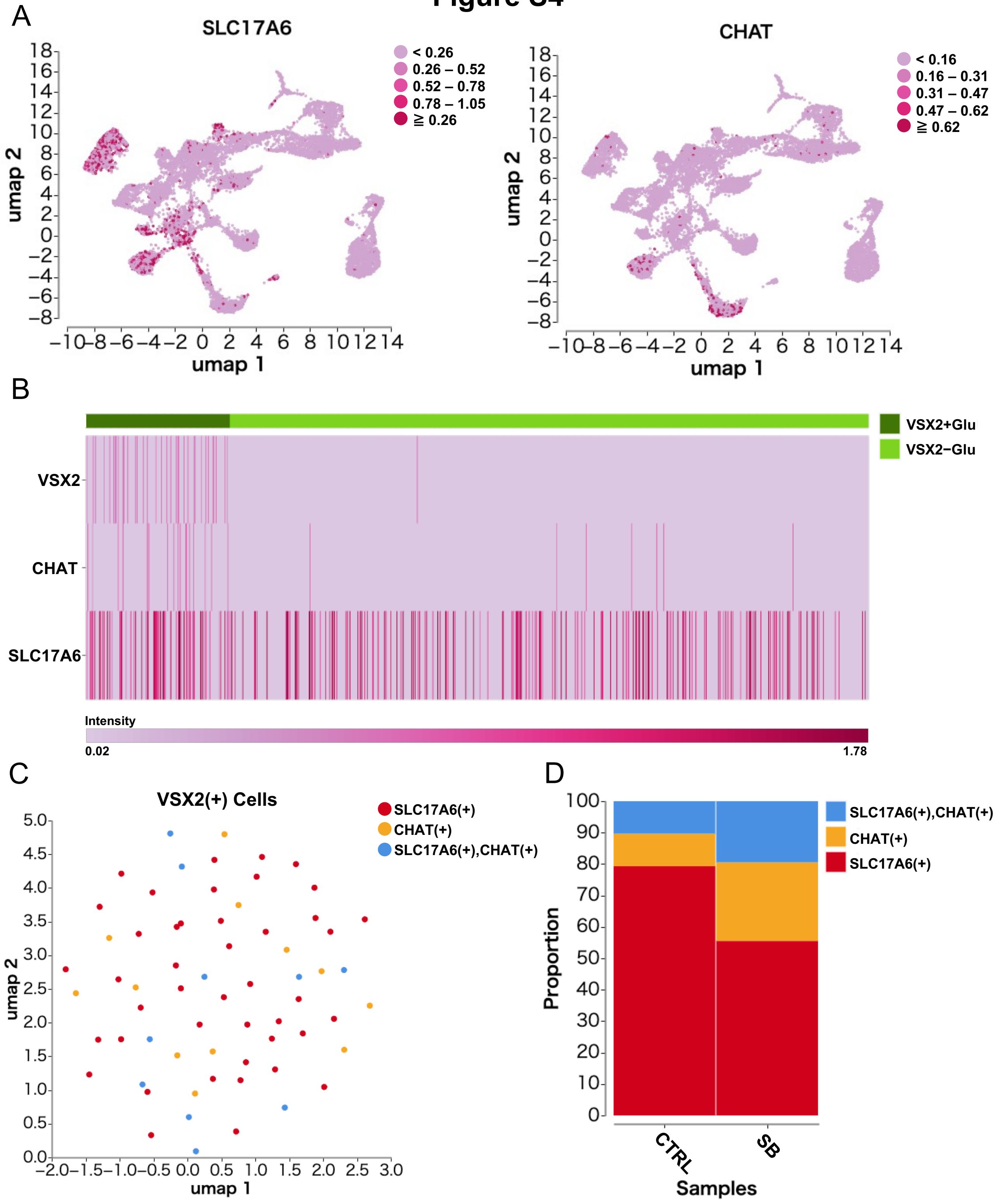
A
SLC17A6
umap 2
umap 1
< 0.26
0.26 – 0.52
0.52 – 0.78
0.78 – 1.05
≧ 0.26
CHAT
< 0.16
0.16 – 0.31
0.31 – 0.47
0.47 – 0.62
≧ 0.62
B
VSX2+Glu
VSX2−Glu
VSX2
CHAT
SLC17A6
Intensity
0.02
1.78
C
VSX2(+) Cells
SLC17A6(+)
CHAT(+)
SLC17A6(+),CHAT(+)
umap 2
umap 1
D
Proportion
Samples
CTRL
SB
SLC17A6(+),CHAT(+)
CHAT(+)
SLC17A6(+)

Figure S1. Neural induction from human induced pluripotent stem cells (hiPSCs).
(A) Schematic of the neural differentiation protocol. Numbers under the line show the days in culture. CTraS stands for Chemically Transitional EB-like State, which is induced by dorsomorphin, SB431542, and CHIR99021 to enhance neural differentiation [1].
(B) Phase-contrast images obtained at various differentiation stages in vitro. Scale bar represents 50 μm.

Figure S2. Enrichment of calcium signaling pathways in the bulk RNA-seq dataset.
(A) Pathview visualization of the results of differentially expressed gene (DEG) analysis in the KEGG pathway map of the calcium signaling pathway (hsa04020). Green indicates transcripts that are downregulated in sleep bruxism (SB), whereas red indicates transcripts that are upregulated in SB.
(B) List of transcripts with significant upregulation or downregulation in the calcium signaling pathway associated with SB. Gene ID, Gene symbol (gene_sym), log2FoldChange(SB/CTRL), p-value (pvalue), and adjusted p-value (padj) of each gene are shown in the list.

Figure S3. Details of neural cell type annotation in the snRNA-seq data.
(A) UMAP visualization showing the distribution of cells across 16 clusters using the Louvain method.
(B) UMAP embedding with annotated clusters, where neurons and progenitor cells were identified based on the expression of specific marker genes. One cluster remained unclassified due to the absence of definitive marker gene expression for specific cell types.
(C) Heatmap displaying the expression levels of various marker genes used for the annotation.

Figure S4. Visualization of *SLC17A6* and *CHAT* expression in glutamatergic neuronal clusters.
(A) UMAP visualization of *SLC17A6* and *CHAT* gene expression. Cells with low expression levels are shown in light pink, whereas cells with higher expression levels are displayed in progressively darker shades.
(B) Heatmap visualizing the expression levels of *VSX2*, *CHAT*, and *SLC17A6* in glutamatergic neuronal subclusters.
(C) UMAP visualization of VSX2(+) neurons classified by gene expression profiles. The colors represent the expression of neuronal subtype-specific genes *SLC17A6* and/or *CHAT*.
(D) Bar plot comparing the proportions of neuronal subtypes within VSX2 (+) neurons in CTRL and SB samples. The stacked bar graph shows the relative abundance of three neuronal subsets. These data suggest that VSX2(+) neurons exhibit characteristics of both glutamatergic and cholinergic neuronal subtypes.

Reference
[1] Fujimori K.; Matsumoto T.; Kisa F.; Hattori N.; Okano H.; Akamatsu W. Escape from pluripotency via inhibition of TGF-β/BMP and activation of Wnt signaling accelerates differentiation and aging in HPSC progeny cells. *Stem Cell Rep*. 2017, 9, 1675–1691 [doi:10.1016/j.stemcr.2017.09.024].
